# The Frail’BESTest: an adaptation of the “balance evaluation system test” for frail older adults; Concurrent validity, responsiveness, validity for fall prediction and detection of slower walkers

**DOI:** 10.1186/s11556-021-00276-8

**Published:** 2021-10-28

**Authors:** A. Kubicki, D. Laroche, L. Coquisart, G. Basile, M. Brika, F. Mourey

**Affiliations:** 1 INSERM UMR1093-CAPS, Université Bourgogne Franche-Comté, UFR des Sciences du Sport, F-21000 Dijon, France; 2 UFR Santé, Université de Bourgogne Franche-Comté, 4 place Tharradin, 25200 Montbéliard, France; 3 INSERM CIC 1432, Plateforme d’Investigation Technologique, University Hospital of Dijon, 23A rue Gaffarel, 21000 Dijon, France; 4 Centre Hospitalier Durécu-Lavoisier, 116 Rue Louis Pasteur, 76160 Darnetal, France

**Keywords:** Systemic assessment, Balance, Frail older adults, Rehabilitation

## Abstract

**Background:**

The Frail’BESTest was developed in order to include frail older adults when they are using the BESTest. Recently, psychometrics properties (internal coherence, systems usefulness, complementarity and inter-rater reliability) of the Frail’BESTest were tested. To complete these analyses, this study will aim the assessment of its concurrent validity, responsiveness, predictive validity on falls occurrence, and slower walkers detection.

**Methods:**

The correlation between the Frail’BESTest and the Gait Speed Test permitted to assess concurrent validity. The variation between the initial test score and the score obtained after the completion of a rehabilitation program was used to evaluate responsiveness with MANOVA analysis and standard response mean (SRM) calculation. Predictive validity was assessed with receiver-operating characteristic curves and area under the curve (AUC) analysis regarding falls occurrence. Slower walkers detection thresholds were computed by receiver-operating characteristic curves for the Frail’BESTest and the Tinetti test.

**Results:**

The concurrent validity of the test was good (r = 0.74; *p* < 0.001). The Standard Error of measurement was at 2.81 points and the Minimal Detectable Change at 7.79 points for the total score of the Frail’BESTest. The SRM was at 0.41 for the Tinetti test and 0.56 for the Frail’BESTest. The AUC, computed according to fall occurrence, was at 0.71 for the Gait Speed test, 0.673 for the Tinetti test and 0.693 for the Frail’BESTest. Both the Tinetti (AUC = 0.87) and the Frail’BESTest (AUC = 0.88) were found suitable for tracking slower walkers.

**Conclusion:**

Concurrent validity and responsiveness of the Frail’BESTest were good. As for the Tinetti and the Frail’BESTest, they were unable to predict efficiently falls occurrence in the tested sample. The Frail’BESTest seems enough sensitive to spot the slower walkers efficiently, using a 15/20 threshold method. The Frail’BESTest was found to be a valid and responsive clinical test, therefore it can be recommended as an outcome measure in clinical practice.

## Introduction

In 2009, Horak et al. proposed a clinical balance assessment tool (called the BESTest) that aims to target 6 different balance control systems (biomechanical constraints, stability limits, transitions- anticipatory postural adjustment, reactive postural response, sensory orientation, stability in gait) in order to design distinct balance deficits through specific rehabilitation approaches. Although functional tests identify which patients may benefit from balance retraining, they do not help therapists build their rehabilitation program, specifically treating the balance problems. The BESTest was developed to support physical therapist identifying the underlying postural control systems that could be involved in the balance deficit so that treatments can spot the abnormal underlying systems. The theoretical framework for developing this test that separates control of balance into its underlying system was based on several postural disorders analysis and medical education of Horak and Shumway-Cook between 1990 and 1999 [1–4].

The Frail’BESTest is a modified version of the BESTest [5] that is dedicated to the systemic assessment of frail older adults. The Frail’BESTest, which was presented in a first paper [6], aims to assess the different and complementary systems used for motor functions in order to optimize the rehabilitation treatment program. The Frail’BESTest is an adapted version of the BESTest that will make it practical to use in frail older adults. Our analysis of the Frail’BESTest protocol measured the usefulness of each system complementarily, and demonstrated the inter-rater reliability of the test. Further assessments were needed to continue the validation process of the Frail’BESTest. Firstly, concurrent validity is important to confirm that the evaluation will be carried out in the same manner as a well-validated tool frequently used for this type of measurement. In the Frail’BESTest, a systemic approach was developed to assess balance function based on the BESTEST model. Then, this test remained an evaluation of balance in older adults. However, the different items are organized into systems, related to specific motor abilities, in order to guide the therapist during the assessment. Therefore, it is particularly important to compare the Frail’BESTest with another well-validated/gold standard test to assess balance function in older adults.

Responsiveness is also a highly relevant measure of the effect of a therapeutic intervention like a physiotherapy program. Therefore, any test dedicated to patient assessment in physiotherapy should demonstrate a good responsiveness. Ideally, the test should be able to detect test – retest changes [7].

The Frail’BESTest was designed specifically for the assessment of the frail older adults. Falls and its consequences represent an important public health issue for older adults in the western countries [8], and could be considered as a multi-factorial phenomena resulting from disturbances in balance function, cognitive function, social conditions, the daily living environment, nutritional status, and other factors [4, 5]. Among these many causes, functional status (including balance) seems to be a key factor [9]. In a recent review, Park and Lee showed that The Berg Balance Scale (BBS) is a suitable tool for examining the risk of falls [10]. Moreover, the Mobility Interaction Fall chart has also interesting results for predicting falls in older adults and frail older adults [11]. Nonetheless, the Tinetti test is widely used to predict falls in clinical practice, because it is thought to be an interesting predictor of falls in older adults, although that would not be enough to complete the analysis [12, 13]. In this context, it seems necessary to confirm the potential usefulness of the Frail’BESTest, its ability to assess balance in frail older adults, to detect some potential changes accurately, to predict future falls and to detect the slower walkers. This paper aims to complete the assessment of the Frail’BESTest by checking the concurrent validity, the responsiveness and the predictive capacities of the test. The Tinetti test and the gait-speed (GS) test, which are widely used in clinical practice in France, contrary to the above-mentioned tests, were used as gold standard throughout our analyses. The Tinetti test (Performance Oriented Mobility Assessment) [14] has proved its reliability in institutionalized aged adults with interrater reliability coefficients ranging from 0.80 to 0.95 and reported test-retest reliability from 0.72 to 0.86 [15, 16]. The Tinetti test has also exhibited construct validity with gait speed in people with Parkinson disease and with the Timed Up and Go in older adults [16, 17]. The GS test required a subject to walk 10 m at normal speed, with a 1-m start-up before timing starts, and a stop order given after the finish line [18]. The GS score was used to predict hospitalization [19, 20], functional and health decline 22], as well as the occurrence of falls [21, 22]. The GS score could also be used to predict a reduction in mortality in older adults [23].

## Methods

### Context, methods and participants

**Fig. 1:**
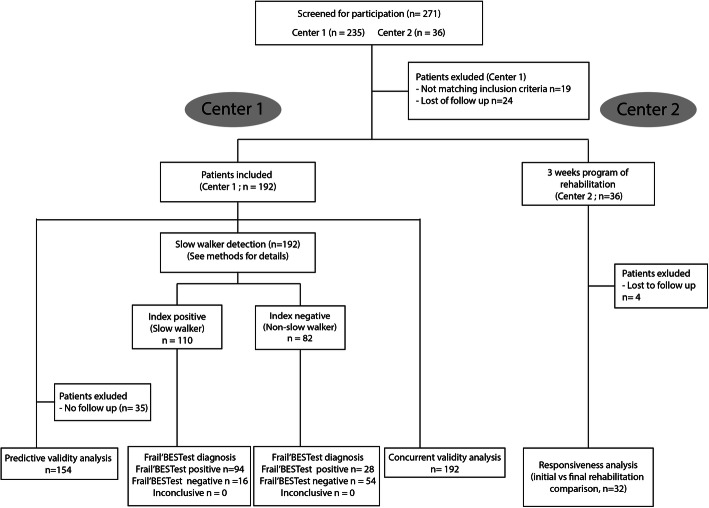
Flow chart for recruitment in center 1 and center 2

We enrolled 192 patients at center 1 and 36 patients at center 2, aged 67 to 95 years; 65.7% of the population was female. The inclusion criteria were as follows:

Patients were initially recruited from a French geriatric department (center 1) and then from a second geriatric department in another region (center 2). Table 1 displays the baseline characteristics of patients in terms of frailty, age, Body Mass Index (BMI), Activity of Daily Living (ADL) and Instrumented Activity of Daily Living (IADL) [24] and motor tests (Gait Speed; Tinetti score; Mini Motor test score and Frail’BESTest scores). The threshold of 0.65 m.s-1 in the gait speed test was used to detect the physical frailty state. Although it is not the only usable criteria, the gait speed has proved to be a very good landmark regarding physical frailty and severe outcome [25–27]. Figure 1 displays patient’s distribution in the two centers (Flow diagram).

**Table 1:** Baseline characteristics of patients

Parameters	Not Frail Mean (SD)	Frail Mean (SD)	***P***-Value
**Male / female**	35 / 51	45 / 104	*P* = 0.10
**N**	86	149	
**Age (years)**	82 (5.4)	85.3 (4.7)	P < 0.001
**BMI**	26.6 (5)	27.1 (5.2)	*P* = 0.47
**ADL Score**	5.5 (0.6)	4.7 (1.3)	P < 0.001
**iADL Score**	3.8 (2.5)	3 (2.3)	P < 0.01
**MMSE**	20.5 (5.1)	19.5 (5.4)	P = 0.10
**Gait Speed (m/sec)**	0.8 (0.1)	0.5 (0.1)	P < 0.001
**Tinetti Score**	23.9 (3.3)	17.2 (3.9)	P < 0.001
**Frail’BESTest Score**	20 (3.6)	12.8 (4.6)	P < 0.001
**Mini-motor Test**	18.6 (1.7)	14.3 (3.4)	P < 0.001

Patient data were collected to be used in the following analyses: I. concurrent validity, II. responsiveness, III. predictive validity of falls and IV. Slower walkers detection.

The local ethics committee of the François Mitterrand hospital approved the experimental protocol, which was carried out in agreement with legal and international requirements (Declaration of Helsinki, 1964). Patients were recruited in center 1 during their outpatient consultation after they had provided informed consent. The motor evaluation was done by an experienced physiotherapist before any others evaluations in this center. The motor evaluation included the Frail’BESTest [6] and the Tinetti test [14]. The results of these two tests were compared to calculate concurrent validity on a population of 192 patients. At a mean of 6.4 ± 1.8 months after the first evaluation, 154 patients were assessed a second time. The results were used to construct concurrent receiver operating characteristic (ROC) curves to assess the predictive validity of the two tests (the Frail’BEStest and the Tinetti test). In order to establish accurately fall prediction, it was essential to determine the definition of a fall. Fall was defined as “an unexpected event in which the participant comes to rest on the ground, floor, or lower level” [18]. As recommended by Ballinger and Payne [20], patients were asked the following: “have you had any fall including a slip or trip in which you lost your balance and landed on the floor or ground or lower level?”

In center 2, the recruited patients were assessed during the first session of their rehabilitation program. The motor evaluation included the Frail’BESTest and the Tinetti test, and was conducted at the beginning of the first session for all included participants. The Frail’BESTest was the only supplementary test done specifically for the study. The data from the first and the second evaluation were used to calculate the responsiveness of the two tests.

In both centers, the exclusion criteria were the inability to stand up with help and the inability to understand the therapist’s instructions. Thirty-two patients completed the full program and were further analyzed.

### Material

**Fig. 2:**
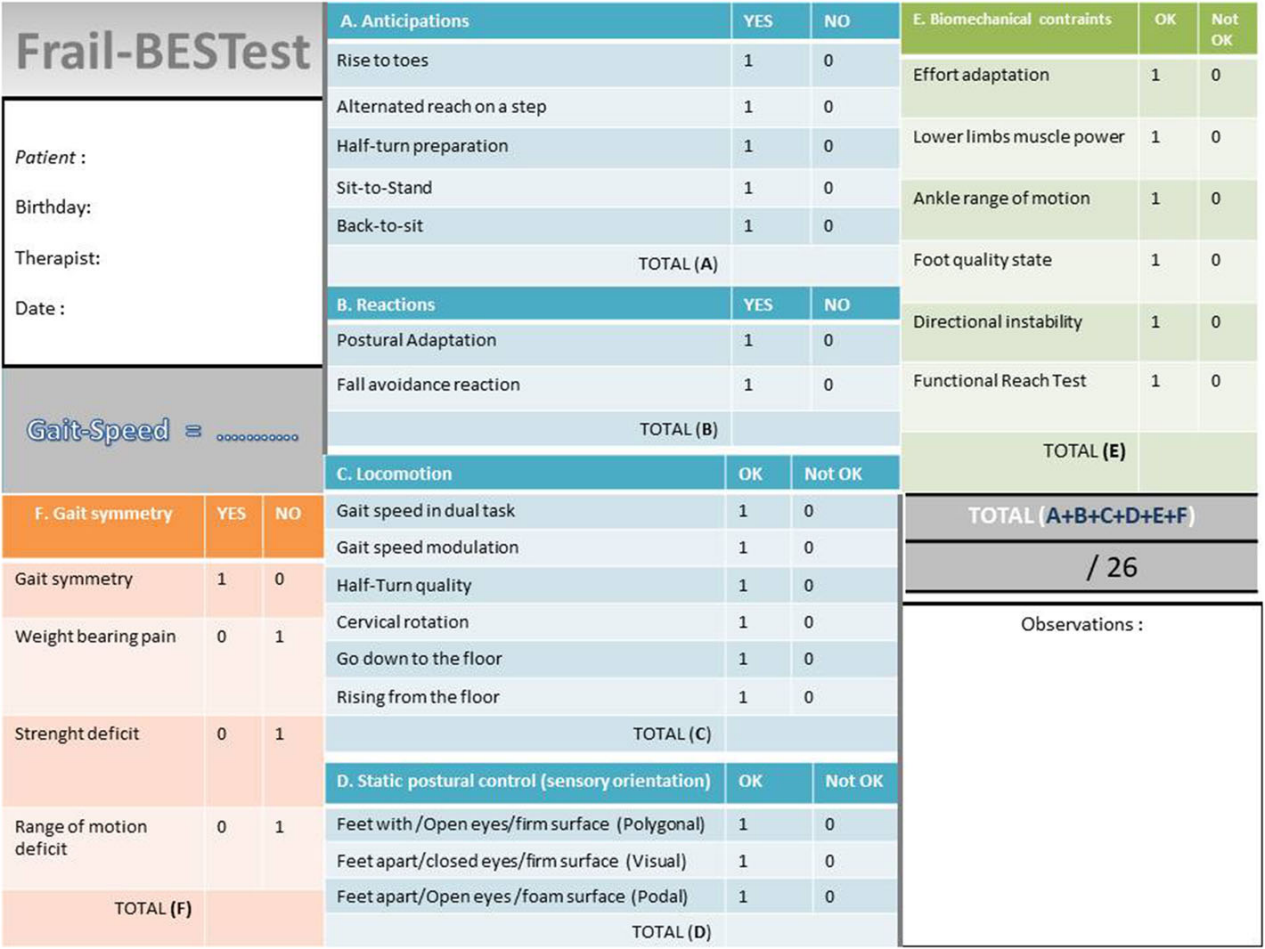
Details of the domains of The Frail’BESTest [2]

The Frail’BESTest has been validated in a previous study [6]. It was built to identify the disorders underlying motor control. Higher total score indicates better functions. Therapists can therefore directly manage therapeutic intervention for different types of motor deficiencies. As shown in Figs. 2, 6 sub-systems have been addressed: A: anticipations, B: reactions, C: locomotion, D: sensorial orientation, E: biomechanical constraints and F: asymmetric gait.

The motor assessments were done either in a large corridor (for gait analysis) or in the physiotherapist’s office (for the other evaluations). The Frail’BESTest required only two specific devices: foam (mean density 55 kg/m3) for the “sensorial preferences” section and a 12-cm-high rehabilitation step for the “anticipations” section. Motor assessments were immediately noted by the physiotherapist and entered into a specific computer file.

### Statistical analysis

Mean and standard deviations were computed for each variable. Statistical analyses were performed using SPSS (IBM Software, NY, USA, version 20) and Stata (The Statsoft, TX, USA, version 15). A significance threshold of *p* < 0.05 was adopted. The strength of correlations was based on Munro’s correlation descriptors (very low = 0.15–0.24, low = 0.25–0.49, moderate = 0.50–0.69, high = 0.70–0.89, and very high = 0.90–1.00).

#### Concurrent validity (center 1)

This analysis was performed on 192 patients. The strength of the correlation between the Frail’BESTest and the Gait Speed Test (GS test) was used for concurrent validitymeasured by the Pearson correlation coefficient. We considered a correlation coefficient of 0.7 as the threshold for validity.

#### Responsiveness (center 2)

This analysis was performed on 32 patients. Standard Error of Measurement (SEM) and Minimal Detectable Change (MDC) were measured from inter-rater reliability data calculated from the first paper about the Frail’BESTest [6]. We proceeded as follow [28]:

The SEM was chosen to test absolute reliability and to represent the absolute error of a measurement. The following formula was used:

1 $$ SEM={\delta}^2\times \sqrt{1- Reliability} $$

where δ^2^ the standard deviation of the measurement and *Reliability* measured by the Kendall’s Tau (Table 2). SEM also expressed relative to the mean of the two measures (%).

**Table 2:** Reliability (Kendall’ Tau), Standard Error of Measurement (SEM) and Minimal Detectable Change (MDC) for the Frail’BESTest

***Frail’BESTest***	Kendall’ Tau	SEM	MDC
***TOTAL***	0.76	2.81	7.79
***Anticipations***	0.73	0.94	2.61
***Reactions***	0.9	0.26	0.73
***Locomotion***	0.83	0.78	2.16
***Static postural control***	0.65	0.64	1.78
***Asymmetric gait***	0.74	0.46	1.28
***Bio-mechanical constraints***	0.89	0.45	1.24

o Minimal detectable change (MDC).

This parameter addressed the problem of deciding whether the result was significant or not. It defined the absolute or relative change that was not due to the variation in the measurement. It was computed in absolute or relative terms using the follow formulae, respectively:

2 $$ MDC= SEM\times 1.96\times \sqrt{2} $$

where SEM was calculated with the eq. (1) above.

The difference between the test scores before and after a rehabilitation program completed in center 2 was used to evaluate the responsiveness of the Frail’BESTtest. This difference was measured with a MANOVA between final and initial values and standardized responses means (SRM). SRM was computed by calculating the mean difference between the pre and post-evaluations divided by the standard deviation of the mean difference. The magnitude of the difference was considered small (0.2 < SRM ≤ 0.5), moderate (0.5 < SRM ≤ 0.8), or large (SRM > 0.8) [29].

#### Predictive validity (center 1)

This analysis was performed on 154 patients due to 35 patients lost to follow up. Predictive validity assessments are usually made with ROC curves. ROC curves provide a cutoff-independent method for the evaluation of continuous or ordinal tests used in clinical assessment. The area under the curve (AUC) is a useful overall measure in test accuracy and can be used to compare different tests (or different equipment) used by the same tester [30]. A higher AUC indicates a higher predictive validity.

#### Detection of slower walkers (center 1)

This analysis was performed on 192 patients. A threshold of 0.65 m.s^− 1^ [31] in the gait speed test was used to determine sensitivity and specificity of the test to detect slower walkers, because it represents a major component of physical frailty. There were multiple logistic regressions carried out for the Frail’BESTest and the Tinetti Test. For each of them, the dependent variable was the gait speed (binary yes/no variable for velocity under or higher or equal 0.65 m/s) and the independent variables were the Frail’BESTest or the Tinetti test with age, sex and ADL score as covariates. ROC curves were done with parameters judged significant in the previous regression and the area under the curve were calculated and compared.

#### Clinical tests comparison (center 1)

To complete the analysis, we evaluate the relationships between Frail’BESTest of 192 patients to other clinically validated tests: the Timed Up and Go test (TUG) [32], the Tinetti test [14] and the Mini-motor test (MMT) [33].

## Results

### Concurrent validity

**Fig. 3:**
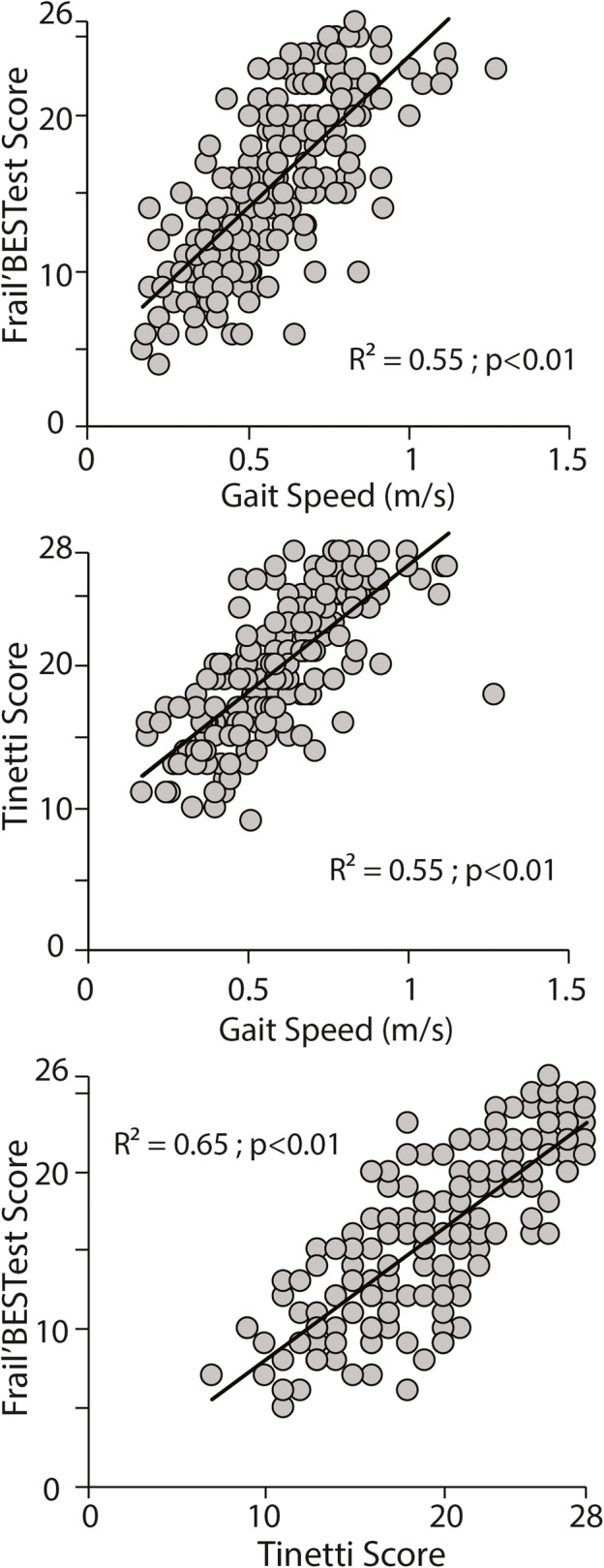
Concurrent validity between each pair of tests in the 192 patients evaluated at center 1

The Frail’BESTest was found to have good concurrent validity (R^2^ = 0.55; *P* < 0.01 against the GS test) and (R^2^ = 0.65; P < 0.01 against the Tinetti test). A comparison of Tinetti’s scores versus GS scores also revealed good concurrent validity (R^2^ = 0.55; P < 0.01) (Fig. 3). No significant differences were found between these correlation coefficients (*P* > 0.11). Frail’BESTest was also correlated with the Tinetti Test (r = 0.80, P < 0,001) and the mini-motor test (r = 0.78, P < 0,001). These two correlations were not significantly different (*P* = 0.60). Please see these results on Fig. 3.

**Fig. 4:**
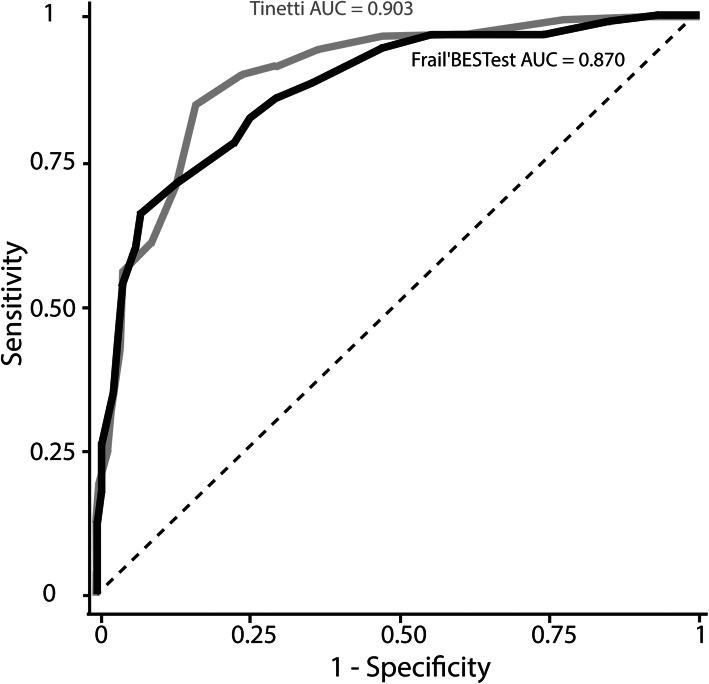
ROC curves of the Frail’BESTest (black) and the Tinetti (grey) to diagnose frailty

### Responsiveness

SEM and MDC are presented in Table 2.

Between the initial and the final evaluation (center 2), both the Tinetti scores (*P* = 0.026) and the Frail’BESTest scores (*P* = 0.003) increased significantly. The Tinetti score (28 points maximum) increased from 18.46 ± 5 initially to 20.36 ± 5.32. The Frail’BESTest score (26 points) increased from 15.89 ± 5.62 initially to 18.45 ± 5.66. The SRM were at 0.41 for the Tinetti test and 0.56 for the Frail’BESTest. See Table 3 for results. The effect sizes for the MANOVA were calculated as partial eta squared (ηp^2^). The statistical power for these analyses was also reported in the table.

**Table 3:** Mean and standard deviations for initial and final evaluations of the Tinetti test and the Frail’BESTest. *P* value, η_p_^2^, statistical power and standard response mean (SRM) are indicated relative to the improvement in the score for both tests

	Initial score (Mean ± SD)	Final score (Mean ± SD)	P value (Scheffé)	ηp^**2**^	Statistical power	SRM
**Tinetti test**	18.4 ± 5	20.3 ± 5.3	0.09	0.04	0.39	0.41
**Frail’BESTest**	15.8 ± 5.6	18.4 ± 5.6	0.03	0.06	0.56	0.56

### Predictive validity

The AUC for the three scores (Gait Speed, Tinetti test, Frail’BESTest) were computed relative to the occurrence of falls at a mean of 6.43 ± 1.8 months after the first evaluation in the 154 patients who completed this second evaluation. Overall, 36% of the participants fell between the first and the second evaluation. The AUC of the Gait Speed test, Tinetti test and Frail’BESTest were at 0.71; 0.673 and 0.693, respectively.

### Slower walkers’ detection

Frail’BESTest and Tinetti test were the only significant variables among sex, age and ADL score in their respective multiple logistic regression (*P* < 0.001 for Frail’BESTest and Tinetti and *P* > 0.11 for other variables). The odds ratio for the detection of slower walkers for the Tinetti (0.63 [0.56–0.71]) and Frail’BESTest (0.70 [0.64–0.76]) were significant (P < 0.001). We then carried out ROC analysis for Frail’BESTest and Tinetti test independently. The AUC were 0.903 [0.859–0.947] for the Tinetti and 0.870 [0.818–0.919] for the Frail’BESTest. The ROC curves for the Frail’BESTest (black) and the Tinetti (grey) were not significantly different (*P* = 0.11) and were plotted in Fig. 4. For the Frail’BESTest, a threshold of 15/26 led to a detection of slower walkers with a sensitivity of 85.1% and a specificity of 65.6%. A threshold of 21/28 on the Tinetti test was used to detect slower walkers with a sensitivity of 80.5% and specificity of 83.6%.

### Clinical tests comparison

Frail’BESTest scores correlated significantly with Tinetti test scores (r = 0.80; *P* < 0.001) TUG test (r = − 0.61; P < 0.001) and with MMT scores (r = 0.78; P < 0.001).

## Discussion

The aim of this paper was to complete a comprehensive assessment of the Frail’BESTest. A first paper checked the contribution of each system, the internal consistency, the threshold and ceiling effects and the inter-rater reliability [6]. The results showed the contribution of each system to the total score of the test by means of an ANOVA. The internal consistency was moderate to good for five systems and limited for one of them. The distribution of the Frail’BESTest was more centered than the Tinetti and Mini-Motor tests. The inter-rater reliability (measured by Kendall’s tau) was strong in the first center and moderate in the second center. Moreover, the Frail’BESTest is easy to follow in clinical practice with an experienced physiotherapist. The test needs eight to ten minutes to complete with a mild-impaired patient. In accordance with the BESTtest theoretical framework, the Frail’BESTest prioritize the patient needs, identifying the most challenging systems. Thus, prioritization is an important matter in a geriatric context due to patient’s fatigability. Such choice involves the most efficient exercises in order to improve the rehabilitation outcomes.

Our results indicate that the concurrent validity of the Frail’BESTest was good when plotted with the Gait Speed test (R^2^ = 0.55) and the Tinetti test (R^2^ = 0.65) in our population of 192 older adults. In accordance with our hypothesis, the Frail’BESTest was able to measure balance as efficiently as these tests. The Gait Speed test is possibly the most representative functional test for overall motor function; it can effectively discriminate between individuals in an aged population in view of their comorbidities and it can be used to accurately predict patient outcomes [19–23, 25, 26, 34].

Test responsiveness was assessed with a MANOVA test in a population of 32 frail older adults. The results were significant for the Frail’BESTest (*p* = 0.036). The SRM was weak for the Tinetti (SRM = 0.41) and moderate for the Frail’BESTest (SRM = 0.56). These interesting results strongly suggest that the Frail’BESTest is better at measuring changes that occur in a sample of patients following a rehabilitation program. Monitoring functional capacity is an essential part of rehabilitation and accordingly a new dedicated balance test has to be responsive. The Frail’BESTest could facilitate follow-up of balance function in frail older adults by more effectively identifying changes in functional capacity.

However, the Frail’BESTest was not a predictor of future fall in the 154 patients who completed the second evaluation. Only the Gait Speed test reached the threshold of 0.7 for predicting the falls from the 6 to 10 months after the evaluation, with a relatively weak sensitivity (70%). In a recent systematic review and meta-analyses from Park, (2018) [11] the author concluded that the predictive validity of fall risk assessment tools commonly used for older adults are not sufficient. Although the study presents the most sensitive tests, including the Downton Fall Risk Index, Hendrich II Fall Risk Model, STRATIFY, and TUG test [35–37], the author highlights their poor specificity, and the use of only one test to assess fall risk is discouraged. Regarding specificity, Park and Lee suggest that only the Berg Balance Scale (BBS) is a suitable tool to screen for the risk of falls [10]. However, even if the author proposes an inter-study heterogeneity analysis for each test, he does not take into account the differences in the population studied. Indeed, it is easier to detect a potential fall in a general population of older adults than in a population of frail or pre-frail older adults, as most of them will fall in the following years. Moreover, the prediction of falls is not only dependent on motor evaluation. As noticed by several research teams, falls are multi-factorial, integrating several dimensions as balance function, cognitive function, social conditions, daily living environment, nutritional status, and even more [8]. This is another argument suggesting that a test focused on balance alone cannot efficiently detect upcoming falls in a population of frail or pre-frail older adults. We consider that in a frail population it is more relevant to minimize fall risk by preventing sarcopenia, osteoporosis, and motor planning impairments, thus reducing the potential consequences.

The Frail’BESTest was developed to facilitate the inclusion of frail older adults in testing by taking into account their limited functional capacities. In our sample, the Frail’BESTest was able to detect a slower walker, with a threshold of 15/26 points. The Tinetti test was also able to detect slower walker with a threshold of 21/28 points. Although this is not the main objective of these tests, it is interesting to note that both were able to track slower walker, with Frail’BESTest showing better sensitivity (85.1%) and the Tinetti test showing better specificity (83.6%). Accordingly, a negative Frail’BESTest (score up to 15) strongly excludes a diagnosis of frailty based on the gait speed, and a positive Tinetti test (score under 21) strongly suggests frailty based on the gait speed. Since it is important to detect frailty early in order to set up an interdisciplinary care plan, the sensitivity of the Frail’BESTest (i.e. low rate of false negatives) makes it a promising tool for clinical assessment.

The study presents limitations. The psychometric properties of the tests were not evaluated in the same center (responsiveness and concurrent validity), potentially affecting the strength of the results. However, we ensured that there were no discrepancies between the data from the two centers. Some may also wonder whether the BBS or the SPPB would have been a better choice for comparisons. We chose the Tinetti because it is routinely used in France, contrary to the BBS and the SPPB. In addition, it avoids a potential bias by recruiting inexperienced physiotherapists in the study. Further studies could be done to assess more thoroughly the properties of the Frail’BESTest. For instance, it would be interesting to test the tool relative compared to other validated assessment scales, especially the BBS and the SPPB, and to perform complete comparisons more in terms of fall prediction. However, as mentioned above, the Frail’BESTest has been developed to facilitate the exploration of the different systems that contribute to balance function, and not to predict the occurrence of falls. Concerning the fall prediction, we have to mention here a potential recall bias. We asked the 154 patients about their recent history of fall while they came back to the day hospital, at a mean of 6.43 ± 1.8 months after the first evaluation. In case of memory loss in the population of frail aged adults, we could have under-diagnosed the number of falls occurred in this period.

Another limitation could be the absence of multi-criteria frailty diagnosis. The gait speed was mainly used to detect a kind of frailty (based on the gait speed) in this paper, then we had to speak about "slower walkers detection" instead of "frailty detection". As mentioned in the methods, gait speed is a validated outcome to spot the frailty state [25–27]. However, other signs should complete the clinical examination in order to confirm the diagnosis, as the others Fried criteria.

Surprisingly, the measures for responsiveness showed that minimal detectable change of the Frail’BESTest was not reached in the center 2. MDC is a statistical measure that does not take into account the global perception from the patient point of view. Moreover, only 3 weeks of rehabilitation were taken into account to test the responsiveness of the Frail’BESTest. To confirm the usefulness of the Frail’BESTest, further studies need to be increase in the follow-up to validate the persistence of the effect and to determine further psychometrics properties (for example, patient acceptable symptom state, minimal clinical improvement detection, ….).

## Conclusion

Concurrent validity and responsiveness of the Frail’BESTest were good. As the Tinetti, Frail’BESTest was unable to efficiently predict falls occurrence in the tested sample. The Frail’BESTest seems enough sensitive to spot the slower walkers efficiently, using a 15/20 threshold. The Frail’BESTest was also found to be a valid and responsive clinical test, it can be recommended as an outcome measure in clinical practice.

## Data Availability

The datasets used and/or analysed during the current study available from the corresponding author on reasonable request.
